# Elevated Apoptosis and Impaired Proliferation Contribute to Downregulated Peripheral **γ** **δ** T Cells in Patients with Systemic Lupus Erythematosus

**DOI:** 10.1155/2013/405395

**Published:** 2013-08-25

**Authors:** Zhimin Lu, Dinglei Su, Dandan Wang, Xia Li, Xuebing Feng, Lingyun Sun

**Affiliations:** ^1^Department of Rheumatology and Immunology, The Affiliated Drum Tower Hospital of Nanjing University Medical School, Nanjing 210008, China; ^2^Department of Rheumatology and Immunology, The Affiliated Hospital of Nantong University, Nantong 226000, China; ^3^Department of Rheumatology and Immunology, Nanjing First Hospital, Nanjing Medical University, Nanjing 210006, China

## Abstract

*Objective*. To investigate the frequency of peripheral **γ** **δ** T cells in patients with systemic lupus erythematosus (SLE) and its correlation with disease activity and to analyze the apoptotic status, proliferation ability, and intracellular cytokine profile of these cells. *Methods*. Flow cytometry was performed to detect the percentage and intracellular cytokine expression of peripheral **γ** **δ** T cells from SLE patients. Annexin-V/PI double staining was applied to determine the proportion of apoptotic **γ** **δ** and CD3^+^ T cells. **γ** **δ** T cell proliferation was analyzed by CFSE labeling technique. *Results*. The percentage and absolute number of **γ** **δ** T cells were remarkably decreased in active SLE patients compared to those in inactive patients and healthy controls, with **γ** **δ** T cell count negatively correlated with disease activity. Compared with healthy controls, peripheral **γ** **δ** T cells from active SLE patients exhibited higher apoptotic rate and lower proliferation ability, as well as elevated expression of intracellular IFN-**γ**, IL-4, IL-10, and TGF-**β**, but not IL-17 or Foxp3. *Conclusion*. Decreased **γ** **δ** T cells in the peripheral blood of SLE patients might be caused by upregulated apoptosis and downregulated cell proliferation. These **γ** **δ** T cells may secret both pro- and anti-inflammatory cytokines to perform their functions in SLE.

## 1. Introduction 

Systemic lupus erythematosus (SLE) is a typical autoimmune disease involving multiple organs and tissues. Although the pathogenesis of SLE has not yet been fully elucidated, it has generally been accepted that activated T/B lymphocytes and enhanced production of proinflammatory cytokines and autoantibodies can cause damage to specific organs and tissues. Various pro- and anti-inflammatory cytokines, including IFN-*γ*, IL-4, IL-17, IL-10, and TGF-*β*, play crucial roles in the pathogenesis of SLE [1]. 

*γδ* T cells expressing TCR *γδ* chains are a minor population of T cells. Based on different TCR *γδ* chain expressions, human *γδ* T cells can be divided into two subsets: V*δ*1^+^ T cells that are mainly distributed in epithelial and mucosal surfaces, and V*δ*2^+^ T cells that generally coexpress V*γ*9 and exist primarily in the peripheral blood and lymphatic system. In normal human peripheral blood, *γδ* T cells, 70–90% of which are V*γ*9^+^V*δ*2^+^ T cells, account for about 1–5% of total T cells, and they can be activated by small nonpeptide phosphoantigens (e.g., isopentenyl pyrophosphate, IPP) in a TCR-dependent and non-MHC-limited manner [2, 3]. In the early stage of immune responses, *γδ* T cells may bridge innate and adaptive immunity through induction of DC maturation, thus playing important roles in anti-infection, antitumor, and autoimmunity [4–6].

 It has ever been demonstrated that *γδ* T cells play important roles in the development of autoimmune diseases through their capacity of antigen presenting, release of proinflammatory cytokines, interaction with CD4^+^CD25^+^Tregs, and promotion of antibody production by providing B cell help [7]. Increased percentage of *γδ* T cells has been found in the synovial fluids and synovium of patients with active rheumatoid arthritis (RA) [8], and lesions of chronic cutaneous lupus erythematosus displayed the expansion of the V*γ*2/V*δ*2 subset [9]. TCR*β* ^−/−^ MRL/lpr mice developed a moderate disease while TCR*δ* ^−/−^ MRL/lpr mice showed exacerbated renal disease and increased mortality, indicating that *γδ* T cells may participate in the development of lupus [10]. The number of *γδ* T cells was abnormal in the peripheral blood, skin, and panniculus of SLE patients [11, 12], but the precise role of *γδ* T cells in the pathogenesis of SLE remains elusive.

In this study, we aimed to investigate the distribution of *γδ* T cells in the peripheral blood of SLE patients and its relation to disease activity and to analyze the apoptotic status, proliferation ability, and intracellular cytokine profile, including IFN-*γ*, IL-4, IL-10, IL-17, and TGF-*β*, in these *γδ* T cells.

## 2. Materials and Methods

### 2.1. Patients and Controls

Forty-two SLE patients fulfilling the 1997 SLE classification criteria revised by American College of Rheumatology (ACR) [13] and 20 age- and sex-matched healthy controls (HC) were enrolled in this study. All patients were not complicated with infection, tumor, or other autoimmune diseases. The median age of these patients was 33 years (range 14–57 years, female/male: 36/6) and that of normal subjects was 30 years (range 18–48 years, female/male: 17/3). The mean score of SLE disease activity index (SLEDAI) was 8.9 ± 3.1 (range 2–16). The mean disease duration was 60.1 ± 57.0 months (range 10 days–252 months). All subjects signed informed consent before the study. Z. M. Lu was responsible for the collection of clinical data and measurement of SLEDAI scores. 

### 2.2. Preparation of Peripheral Blood Mononuclear Cells (PBMCs)

Peripheral blood of SLE patients and healthy controls were collected. 4 mL of heparinized blood was diluted with the same volume of phosphate-buffered saline (PBS). Peripheral blood mononuclear cells (PBMCs) were prepared by Ficoll-Paque (Pharmacia, Uppsala, Sweden) density gradient centrifugation, washed in RPMI 1640 culture medium (Gibco) twice, and then resuspended at a concentration of 2 × 10^6^ cells/mL.

### 2.3. Cell Stimulation and Culture

PBMCs were resuspended in RPMI 1640 medium (Gibco) supplemented with 1% penicillin-streptomycin solution (Gibco) and 10% heat-inactivated fetal bovine serum (FBS) (Gibco). 50 ng/mL of Phorbol myristate acetate (PMA) and 1 ug/mL of ionomycin (Io) (both from Sigma) were added for polyclonal stimuli. 10 ug/mL of Brefeldin A (Sigma) was used for inhibition of cytokine secretion. Samples not stimulated with PMA and Io but exposed to Brefeldin A were served as negative controls. Cells were cultured for 5 h at 37°C in a 5% CO_2_ atmosphere. 

 PBMCs from SLE patients and healthy controls were incubated with 5 *μ*M carboxyfluorescein diacetate succinimidyl ester (CFSE) (Invitrogen) solved in PBS for 10 minutes at 37°C. An excess of ice-cold RPMI 1640 with 10% FBS was added to the cells to quench the reaction. After being washed with RPMI 1640 culture medium twice, these CFSE-labeled cells (1 × 10^6^/well) were cultured in complete RPMI 1640 medium supplemented with 2 mM L-glutamine, 10 mM HEPES, and 10% FBS for 7 days with addition of rhIL-2 (200 U/mL) in the presence or absence of precoated immobilized anti-human *γδ* TCR (1 *μ*g/mL, Biolegend). The proliferation of *γδ* T cells was measured by flow cytometry to determine the CFSE fluorescence intensity. 

### 2.4. Flow Cytometry Analysis

Freshly isolated and cultured PBMCs were suspended in PBS. For the staining of surface antigens, cells were incubated with FITC-conjugated anti-CD3 and APC-conjugated anti-TCR*γδ*. For the detection of intracellular cytokines, cells were stained with appropriate amount of PE-conjugated anti-IL-4, anti-IL-10, anti-IL-17, anti-Foxp3 (all from BD), anti-TGF-*β* monoclonal antibodies (R&D), or PE-Cy7-conjugated anti-IFN-*γ* (BD) for 20 min at 4°C after the fixation/permeabilization process. Foxp3 staining was performed according to the manufacturer's manual. Mouse anti-human FITC-, PE-, and PE-Cy7-conjugated IgG1 were used as isotype controls. All cell samples were assayed by a FACSCalibur flow cytometer (BD Bioscience) and the acquired data were further analyzed using FCS express V3 analysis software. Flow cytometric results were represented as positive percentages or mean fluorescence intensity (MFI). Annexin-V/PI double staining flow cytometry was employed to detect the proportion of the apoptotic *γδ* T and CD3^+^ T cells in 6 active SLE patients and 6 healthy controls.

### 2.5. Statistical Analysis

Data are presented as mean ± standard deviation if not otherwise stated. All data were analyzed using SPSS version 17.0 software. Mann-Whitney *U* test was used to compare means between SLE patients and healthy controls. Pearson correlation analysis was performed to evaluate the correlation between variables of peripheral *γδ* T cells and SLEDAI scores. The *P* values < 0.05 were considered statistically significant. 

## 3. Results

### 3.1. Peripheral *γδ* T Cells Were Reduced in SLE Patients

**Figure 1:**
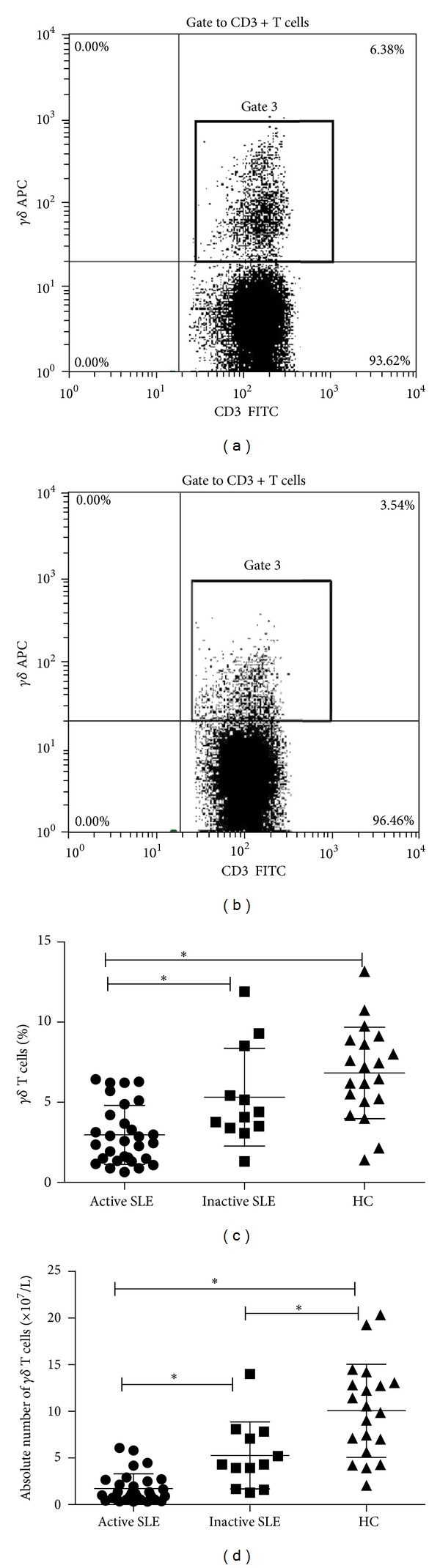
Reduction of *γδ* T cells in peripheral blood of SLE patients. (a) and (b) Representative flow cytometric plot of CD3^+^ *γδ* ^+^ T cells from one healthy control (HC) and one SLE patient, respectively. (c) Comparison of the percentage of peripheral *γδ* T cells among active SLE patients, inactive SLE patients, and healthy controls. (d) Comparison of the absolute number of peripheral *γδ* T cells among active SLE patients, inactive SLE patients, and healthy controls. (*P < 0.01).

We compared the frequency and numbers of peripheral *γδ* T cells between healthy controls and SLE patients. Flow cytometric analysis showed that the percentage of *γδ* T cells was remarkably decreased in active SLE patients (2.96 ± 1.84%) compared to that in inactive patients (5.31 ± 3.05%) and healthy controls (6.83 ± 2.85%, both *P* < 0.01, Figure 1(c)), while there was no difference between inactive SLE patients and healthy controls. The absolute number of peripheral *γδ* T cells was also decreased in active SLE patients ((1.72 ± 1.58) × 10^7^/L) compared to that in inactive patients ((5.27 ± 3.60) × 10^7^/L) (*P* < 0.01), and both of which were lower than that in healthy controls ((10.07 ± 4.99) × 10^7^/L) (both *P* < 0.01, Figure 1(d)). 

### 3.2. Peripheral *γδ* T Cells Were Associated with SLE Disease Activity and Other Clinical Indices

**Figure 2:**
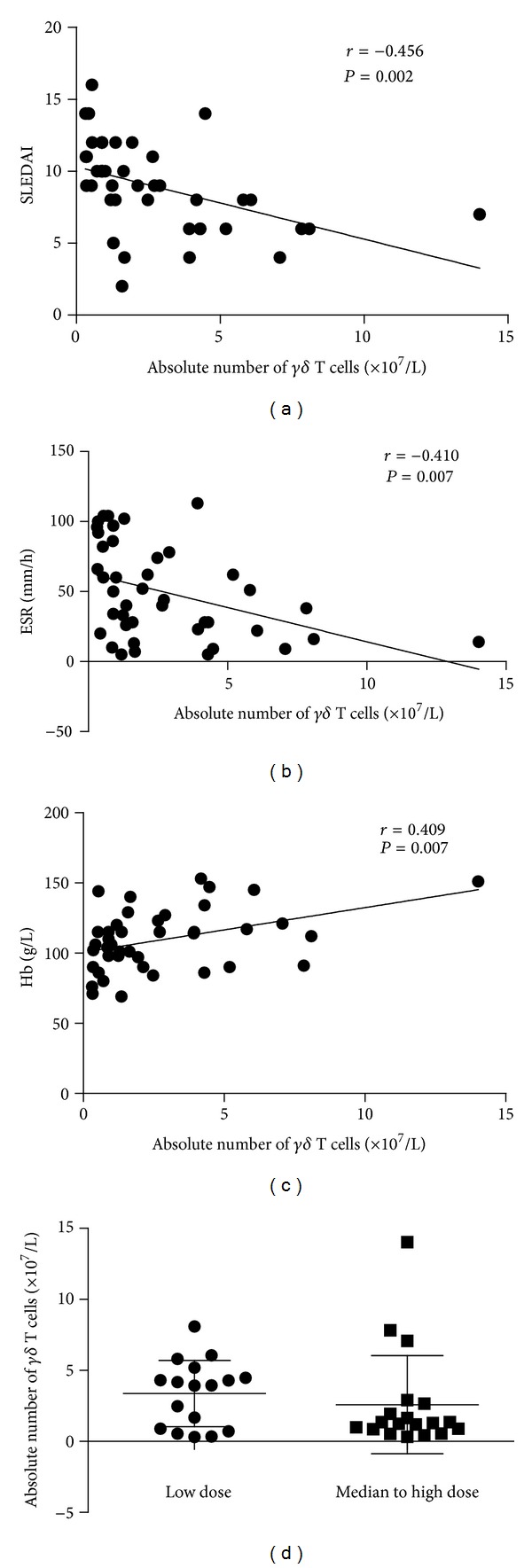
Associations of peripheral *γδ* T cells with SLEDAI, ESR, Hb, and prednisone dosage in SLE patients. (a) Correlation of the absolute number of peripheral *γδ* T cells with SLEDAI; (b) correlation of the absolute number of peripheral *γδ* T cells with ESR; (c) correlation of the absolute number of peripheral *γδ* T cells with serum hemoglobin level; (d), comparison of the percentage and absolute number of peripheral *γδ* T cells with different doses of prednisone.

Correlation analysis showed that in those 42 SLE patients, the absolute number of peripheral *γδ* T cells was negatively correlated with SLEDAI score (*r* = −0.456, *P* = 0.002, Figure 2(a)). The mean blood hemoglobin (Hb) level of all 42 patients was (109.33 ± 21.76) g/L and erythrocyte sedimentation rate (ESR) was 49.60 ± 33.34 mm/h. Our data showed that the numbers of peripheral *γδ* T cells were negatively correlated with ESR level (*r* = −0.410, *P* = 0.007, Figure 2(b)) and positively correlated with Hb level (*r* = 0.409, *P* = 0.007, Figure 2(c)) in SLE patients. As the frequency of *γδ* T cells was reportedly elevated in the normal cutaneous tissue of SLE patients [14], we compared the number of peripheral *γδ* T cells between patients with or without erythema, but no significant difference was observed ((2.11 ± 1.93) × 10^7^/L (*n* = 17) versus (3.16 ± 3.24) × 10^7^/L (*n* = 25)). No significant correlation was found between the number of peripheral *γδ* T cells and the level of serum complement, 24-hour urinary protein, antinuclear antibody (ANA), and anti-dsDNA antibody of these SLE patients. 

We also analyzed the difference of peripheral *γδ* T cell distribution in patients treated with various doses of prednisone. Before recruitment into the study, 17 patients were treated with low-dose prednisone (≤15 mg/d, mean dose (9.4 ± 4.4) mg/d) and 19 patients with median- to high-dose prednisone (>15 mg/d, mean dose (39.5 ± 20.4) mg/d). The number of peripheral *γδ* T cells in these two groups was (3.37 ± 2.32) × 10^7^/L versus (2.58 ± 3.45) × 10^7^/L, respectively, (Figure 2(d)), with no significant difference observed. We recruited additional 6 SLE patients without prednisone treatment, and our data showed no significant difference between the numbers of peripheral *γδ* T cells in patients with or without prednisone treatment ((3.86 ± 2.38) × 10^7^/L (*n* = 6) versus (2.95 ± 2.96) × 10^7^/L (*n* = 36), *P* > 0.05).

### 3.3. Increased Apoptosis and Reduced Proliferation May Account for Decreased Number of Peripheral *γδ* T Cells in Active SLE Patients

**Figure 3:**
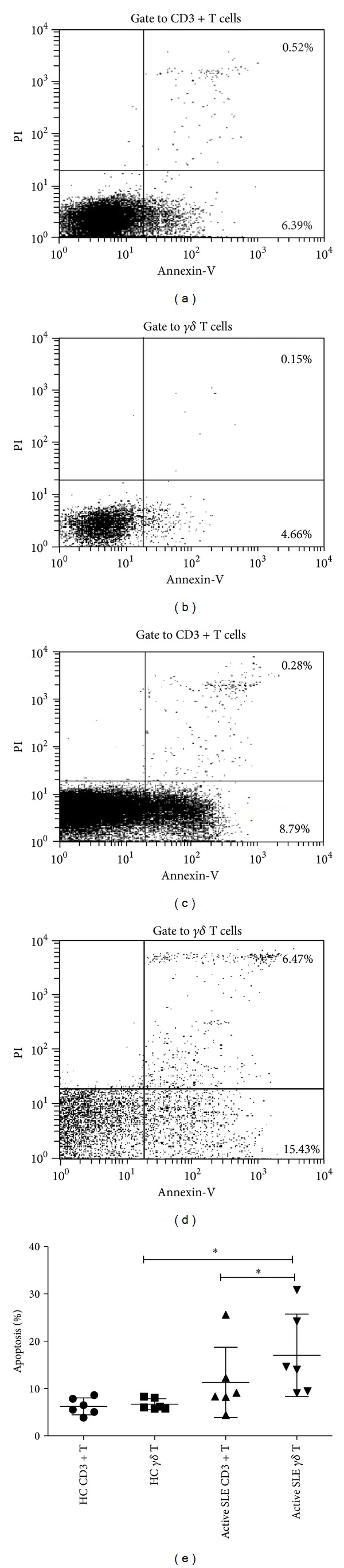
Increased apoptosis of peripheral *γδ* T cells in active SLE patients. (a) and (b) Representative flow cytometric plot for apoptosis of total CD3^+^T cells and *γδ* T cells in one healthy control; (c) and (d) flow cytometric plot for apoptosis of total CD3^+^T cells and *γδ* T cells in one SLE patient; (e) comparison of apoptotic rate of CD3^+^T and *γδ* T cells between healthy controls and active SLE patients (*n* = 6, *P < 0.05).

To delineate the underlying mechanisms for the decrease of peripheral *γδ* T cells in active SLE patients, we analyzed the apoptosis and proliferation status of these cells. AV/PI double staining flow cytometry showed that the apoptotic rate of peripheral *γδ* T cells in 6 active SLE patients was increased compared with that in 6 healthy controls (17.03 ± 8.71% versus 6.67 ± 1.18%, *P* < 0.05, Figures 3(a)–3(d)). The apoptotic rate of *γδ* T cells was significantly higher than that of total CD3^+^ T cells in the peripheral blood of active SLE patients (17.03 ± 8.71% versus 11.30 ± 7.43%, *P* < 0.05, Figure 3(e)), while no significant difference was found in healthy controls (6.67 ± 1.18% versus 6.22 ± 1.78%, *P* > 0.05, Figure 3(e)). 

**Figure 4:**
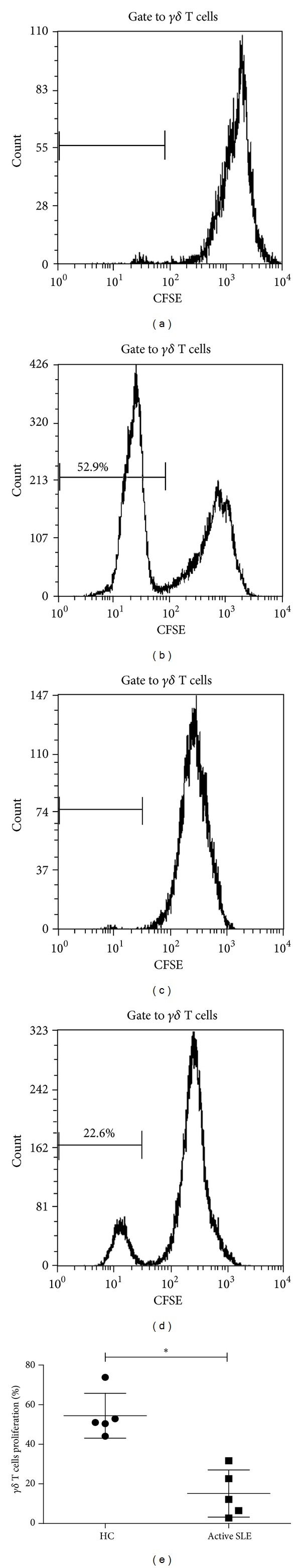
Reduced *γδ* T cell proliferation in active SLE patients. (a) and (b) Proliferation of *γδ* T cells in one healthy control cultured without or with stimulation of rhIL-2 (200 U/mL) and immobilized anti-human *γδ* TCR (1 ug/mL) for 7 days. (c) and (d) Proliferation of *γδ* T cells in a SLE patient cultured without or with stimulation. (e) Comparison of *γδ* T cell proliferation under the stimulation of rhIL-2 and immobilized anti-human *γδ* TCR between active SLE patients and healthy controls (*n* = 5, *P < 0.01).

*γδ* T cell proliferation was determined by CFSE method using PBMC from 5 active SLE patients and 5 healthy controls after in vitro stimulation of immobilized anti-human *γδ* TCR for 7 days. As shown in Figure 4, the proliferation rate of *γδ* T cell in active SLE patients was significantly reduced compared with that in healthy controls (54.43 ± 11.32% versus 15.15 ± 11.90%, *P* < 0.01, Figure 4). 

### 3.4. Peripheral *γδ* T Cells from SLE Patients Expressed Altered Intracellular Cytokine Profile

**Figure 5:**
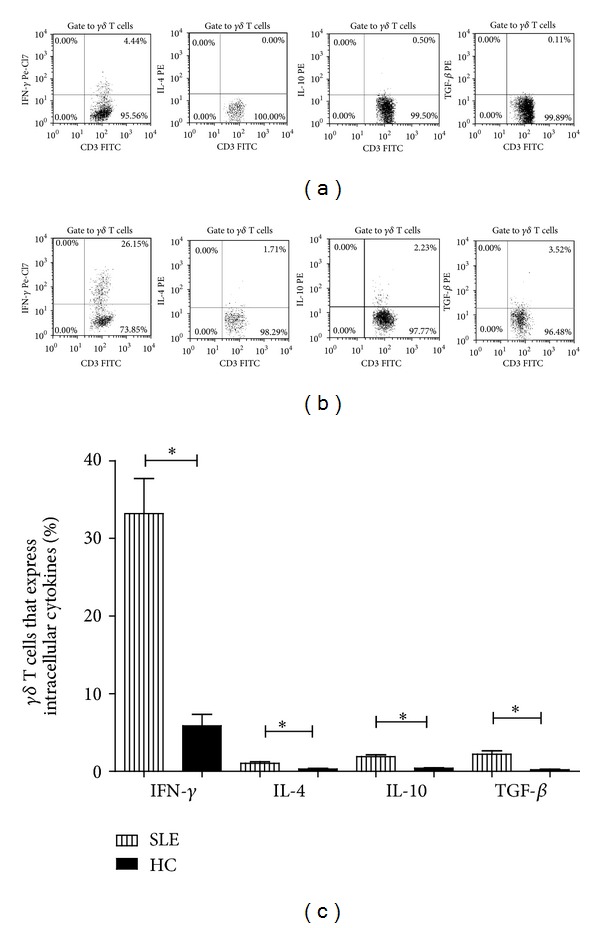
Expression of intracellular cytokines in peripheral *γδ* T cells from SLE patients (*n* = 20, *P < 0.01). (a) Representative flow cytometric plots of *γδ* T cells that express various intracellular cytokines from one healthy control and SLE patient, respectively. The data of IL-17^+^ and Foxp3^+^ *γδ* T cells, which were nearly undetectable, were not shown. (b) Comparison of the percentages of *γδ* T cells expressing IFN-*γ*, IL-4, IL-10, or TGF-*β* in SLE patients and healthy controls.

We examined intracellular cytokine expressions in 20 active SLE patients and 10 healthy controls by flow cytometry. As shown in Figure 5, the percentages of *γδ* T cells that express intracellular IFN-*γ*, IL-4, IL-10, and TGF-*β* in SLE patients (33.19 ± 20.20%, 1.04 ± 0.93%, 1.91 ± 0.98%, and 2.20 ± 1.97%, resp.) were significantly higher than those in healthy controls (5.87 ± 4.63%, 0.30 ± 0.34%, 0.18 ± 0.31%, and 0.21 ± 0.22%, all *P* < 0.01, Figure 5). However, there was no significant difference of the percentage of IL-17^+^ *γδ* T cells and Foxp3^+^ *γδ* T cells between SLE patients and healthy controls (0.14 ± 0.24% versus 0.18 ± 0.31%, and 0.44 ± 0.85% versus 0.49 ± 0.44%, resp., *P* > 0.05). 

## 4. Discussion 

In recent years, more and more attention has been paid upon the role of *γδ* T cells in the pathogenesis of autoimmune diseases including SLE [7, 14]. Our study demonstrated that the number of *γδ* T cells in the peripheral blood of SLE patients was decreased and negatively correlated to disease activity. SLE *γδ* T cells displayed high apoptotic rate, low proliferation ability, and abnormal cytokine profiles as compared to those of healthy controls. 

Though some studies reported an elevated proportion of peripheral *γδ* T cells in untreated SLE [15], the majority of previous reports showed that the percentage and absolute number of *γδ* T cells were reduced in the peripheral blood of SLE patients [14, 16], which was negatively correlated with levels of ESR and C-reactive protein (CRP). Our study confirmed that the percentage of peripheral *γδ* T cells in SLE patients decreased significantly compared with that in healthy controls and was more prominent in those with active disease. The reduced number of *γδ* T cells was negatively correlated with SLEDAI score as well as ESR, suggesting that downregulation of peripheral *γδ* T cells might play a role in the aggravation of SLE disease. 

The underlying mechanism for the decreased number of peripheral *γδ* T cells in SLE patients remains elusive. It has been demonstrated that in patients with chronic cutaneous lupus, *γδ* T cells were enriched in the skin lesions, whereas the percentage of peripheral *γδ* T cells was not changed. Robak et al. also found that the frequency of *γδ* T cells was significantly higher in the normal cutaneous tissue of SLE patients compared with that in healthy controls, and it was reduced after treatment with corticosteroids, which was supported by studies of Spinozzi et al. [15] who had shown that addition of dexamethasone to cultured *γδ* T cells from the peripheral blood of SLE patients could reduce the number of these cells. These results indicated that the reduction in the number of *γδ* T cells could be ascribed to their migration to the skin tissue under the influence of corticosteroids. In this study, we compared the number of peripheral *γδ* T cells from SLE patients with or without skin lesions that of and those treated with different doses of prednisone. There was no significant difference between these groups, suggesting that skin-toward migration and corticosteroid application could not explain the reduction of peripheral *γδ* T cells in our SLE patients. 

Our data showed that the apoptotic rate of *γδ* T cells was higher in SLE patients than in healthy controls, and the frequency of apoptotic *γδ* T cells was higher than that of total CD3^+^ T cells in SLE patients, indicating that the decreased number of *γδ* T cells in the peripheral blood of SLE patients may be partly due to the upregulated apoptosis of these cells. Besides, in vitro culture of peripheral *γδ* T cells from active SLE patients showed reduced proliferation than that from healthy controls under the stimulation of rhIL-2 and immobilized anti-*γδ* TCR, suggesting that the impaired proliferative capacity may also contribute to the decreased number of peripheral *γδ* T cells in SLE patients. 

Apart from abnormal number, *γδ* T cells from SLE patients were also shown to have some functional defects. In this study, we investigated the intracellular cytokine expression of peripheral *γδ* T cells from SLE patients, including IL-4, IL-10, IL-17, IFN-*γ*, and TGF-*β*. Similar to CD4^+^ *αβ* T cells, *γδ* T cells can produce a variety of cytokines in response to different stimulating signals. Cytokines secreted by *γδ* T cells under different microenvironments could influence the immune processes in some diseases, which might play crucial roles in the development of autoimmune diseases [7, 17–20]. Our study showed that in the peripheral blood of SLE patients, the percentages of *γδ* T cells that expressed intracellular IFN-*γ*, IL-4, IL-10, and TGF-*β* were all significantly increased, which was possibly due to the immunological disorder of SLE and in turn might aggravate lupus disease. We further calculated absolute IFN-*γ* expressing cells in samples but found that there was no difference between SLE patients and healthy controls ((0.90 ± 1.27) × 10^7^/L (*n* = 20) versus (0.58 ± 0.53) × 10^7^/L (*n* = 10), *P* > 0.05). To which extent these different subsets of *γδ* T cells exert their pathogenic or protective roles in SLE still needs in-depth investigations in vitro and in vivo. Unexpectedly, although the expression of TGF-*β* was elevated, we did not find significantly increased level of Foxp3 in peripheral *γδ* T cells from SLE patients, suggesting that *γδ* T cells might not exert their immunomodulatory effect through the Foxp3 pathway. 

In conclusion, this study demonstrated that the percentage and absolute number of *γδ* T cells in the peripheral blood of SLE patients decreased substantially compared with those of healthy controls and was conversely correlated with disease activity. The reduction of *γδ* T cells might be attributed to increased apoptosis and impaired proliferative capacity of these cells. Expression of pro- and anti-inflammatory cytokines, including IL-10, IL-4, IFN-*γ*, and TGF-*β*, was significantly increased in the peripheral *γδ* T cells from SLE patients, suggesting that there could be different subsets of *γδ* T cells functioning in the pathogenesis of SLE.
